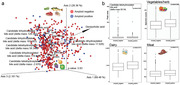# Expanded bile acid annotations linked to diet and AD biomarkers

**DOI:** 10.1002/alz.090001

**Published:** 2025-01-03

**Authors:** Ipsita Mohanty, Andres Mauricio Caraballo‐Rodriguez, Jasmine Zemlin, Jennifer S Labus, Pieter Dorrestein, Rima F. Kaddurah‐Daouk, Alzheimer’s Gut Microbiome Project Consortium (AGMP)

**Affiliations:** ^1^ University of California San Diego, San Diego, CA USA; ^2^ University of California, San Diego, La Jolla, CA USA; ^3^ Goodman‐Luskin Microbiome Center, University of California ‐ Los Angeles, Los Angeles, CA USA; ^4^ Oppenheimer Center for the Neurobiology of Stress and Resilience, David Geffen School of Medicine, University of California Los Angeles, Los Angeles, CA, Los Angeles, CA USA; ^5^ UCLA Vatche and Tamar Manoukian Division of Digestive Diseases, Los Angeles, CA USA; ^6^ Duke Institute for Brain Sciences, Duke University, Durham, NC USA; ^7^ Duke University, Durham, NC USA; ^8^ Department of Medicine, Duke University, Durham, NC USA

## Abstract

**Background:**

Bile acids (BA) are steroids regulating nutrient absorption, energy metabolism, and mitochondrial function, and serve as important signaling molecules with a role in the gut‐brain axis. The composition of BAs in humans changes with diet type and health status, which is well documented with a few known bile acids. In this study, we leveraged a new BA‐specific spectral library curated in the Dorrestein lab at UCSD to expand the pool of detected BAs in Alzheimer‐related LC‐MS/MS datasets and provide links to dietary profiles and AD markers.

**Method:**

Fecal untargeted metabolomics (LC‐MS/MS) data from the ADRC cohort was analyzed using GNPS‐based molecular networking. Spectral matching and annotation were performed using the BA‐specific spectral library which consists of 21,549 BA spectra, with many previously undiscovered candidates. We obtained spectral matches to 113 BAs with 108 matches to new candidate BAs from our library. Further, using the score for delayed recall of Benson figure (UDSBENTD), peak areas of the BAs were plotted with the amyloid status. For diet readout, spectral match to a food database (“global foodomics”) was performed, and correlation with BAs was obtained by joint‐RPCA analysis.

**Result:**

The joint‐RPCA analysis yielded many di‐, tri‐, and tetrahydroxylated BAs among the top 20 features guiding the separation in the ADRC samples (Figure 1a). From diet, meat, fish, and fruits were among the top features, with meat and fish vectors pointing in the opposite direction as grapes, onions, and lettuce. Peak area‐based levels of a candidate tetrahydroxylated BA, which was among the top 20 features, changed with the amyloid status measured with the UDSBENTD (Figure 1b). The spectral count of vegetables and dairy also increased in amyloid‐positive samples. However, the spectral counts related to meat and poultry products did not change significantly with amyloid status, implying a potential vegetable‐based diet impacting the change in some BA levels.

**Conclusion:**

Previously unknown BAs are correlated to diet and AD markers in the ADRC cohort. This study highlights the importance of expanding our metabolite annotations, in this case with BAs, and performing integrative analysis with diet to aid our understanding of AD progression.